# C-reactive protein and high-sensitivity C-reactive protein levels in asymptomatic intestinal parasite carriers from urban and rural areas of Gabon

**DOI:** 10.1371/journal.pntd.0011282

**Published:** 2024-05-20

**Authors:** Helena Noéline Kono, Mérédith Flore Ada Mengome, Bedrich Pongui Ngondza, Roger Hadry Sibi Matotou, Luccheri Ndong Akomezoghe, Bernadette Ekomi, Bridy Chesly Moutombi Ditombi, Jeanne Vanessa Koumba Lengongo, Jacques Mari Ndong Ngomo, Noé Patrick M’Bondoukwé, Cyrille Bisseye, Denise Patricia Mawili-Mboumba, Marielle Karine Bouyou Akotet

**Affiliations:** 1 Department of Parasitology-Mycology-Tropical Medicine, Université des Sciences de la Santé (USS), Owendo, Gabon; 2 Centre de REcherche biomédicale en pathogènes Infectieux et Pathologies Associées (CREIPA), Libreville, Gabon; 3 Laboratoire de Biologie Moléculaire et Cellulaire (LABMC), Université des Sciences et Techniques de Masuku (USTM), Franceville, Gabon; 4 Unité Mixte de Recherche sur les Agents Infectieux et leur Pathologie (UMRAIP), Université des Sciences de la Santé, Owendo, Gabon; University of North Carolina at Chapel Hill, UNITED STATES

## Abstract

**Background:**

Chronic carriage of intestinal parasitic infections (IPIs) can induce chronic inflammation and dysbiosis, which are risk factors for non-communicable diseases. The objective of this study was to determine the relationship between IPI carriage and inflammation in a population of volunteers living in Gabon.

**Methodology and principal findings:**

A cross-sectional study was conducted from September 2020 to November 2021 in asymptomatic volunteers aged 18 years old and over, residing in different areas of Gabon: Libreville (urban area) and Koula-Moutou and Bitam (rural areas). The detection of IPIs was carried out using four common microscopic techniques. C-reactive protein (CRP), and high-sensitivity C-reactive protein (hsCRP) were measured and levels were compared according to the presence or absence of IPI. Overall, 518 participants were included, 64.5% (n = 334) of whom resided in urban area and 35.5% (n = 184) in rural areas. The median age was 35 years (27; 46). The prevalence of asymptomatic IPIs was 29.9% (n = 155), with a significantly higher frequency in rural areas than in urban area (adjusted OR 6.6 (CI 3.2–13.8), *p* < 0.01). Protozoa were more frequent than soil-transmitted helminths (STHs) in both areas: 81.6% (n = 40) in urban area and 69.8% (n = 74) in rural areas. STHs were predominant in rural areas (48.1% vs 22.4% in urban area. In case of IPI, the median values of CRP (15 (13–15) mg/L vs 13.0 (11.1–14.9) mg/L) and hsCRP (4.2 (1.4–13.0) mg/L vs 2.2(0.4–6.1) mg/L) were higher (p<0.01). Elevated hsCRP and CRP were significantly more frequent in parasitized individuals (for hsCRP: 22.6%, n = 35; for CRP: 52.9%, n = 82); in particular among STH carriers (for hsCRP: 65.9%, n = 27, for CRP: 36.6%, n = 15) (*p* < 0.01).

**Conclusions/Significance:**

This first study showed that asymptomatic IPIs, particularly STH carriage are associated with high CRP and hsCRP levels. Further larger and longitudinal studies are needed to elucidate the global and specie-specific enteropathogens link with chronic inflammation.

## Author summary

Repeated or chronic parasitism can maintain local or systemic chronic inflammation, CRP and hsCRP are sensitive biomarkers of subclinical low-grade inflammation. This study assessed the serum levels of CRP and hsCRP in adults with or without intestinal parasite (IPI) carriage according to residence area and parasite species. IPI chronic carriage, especially with pathogenic protozoa and/or STH, was associated with higher levels of CRP and hsCRP. These findings suggest that intestinal parasite carriage contributes to low grade systemic inflammation which is a driver of NCD. The role of chronic carriage of other enteropathogens on chronic inflammation status as well the relationship between IPI and dysbiosis should be further analyzed in endemic countries.

## Introduction

Intestinal parasitic infections (IPIs) due to intestinal protozoa and helminths including soil-transmitted helminths (STHs) still constitute a public health problem in sub-Saharan Africa (SSA). The IPI burden is favored by climatic conditions, the absence or insufficient hygiene and sanitary measures, and poverty [1]. In endemic areas, IPIs are often more frequent in rural than in urban areas, and are generally asymptomatic and undiagnosed, leading to chronic parasite carriage [1]. Thus, chronic parasitism can induce alterations in the gut microbiota, which have been correlated with cardio-metabolic diseases [2,3]. Intestinal infection with Protozoan parasites such as *Cryptosporidium sp*, *Entamoeba (E*.*)*. *histolytica*, *Giardia (G*.*)*. *intestinalis* and *Blasctocystis sp* are associated with altered microbiome profile and/or decreased abundance of the gut commensal bacteria, leading to gut dysbiosis [4–6]. Chronic disruption of the gut barrier can lead to translocation of microbial components into the body, producing systemic, low-grade inflammation [7]. Likewise, *Blastocystis hominis* and *Giardia intestinalis* have been shown to induce local inflammatory responses which are other causes of dysbiosis [8,9]. Cardiovascular diseases (CVD), type 2 diabetes, and metabolic syndrome are associated with persistent or chronic inflammation, even low-grade, by inducing or increasing insulin resistance and atherosclerosis [10,11]. Thus, while diet and smoking are more frequently associated with the occurrence of non-communicable diseases (NCD) in high-income countries, with NCD-related mortality associated with older age groups, other factors such as younger age, could be a cause in low- and middle-income countries (LMIC) where NCD related deaths frequently occur in 30-69-year-old individuals [12]. This age group is also frequently exposed to intestinal parasite chronic carriage in endemic areas [13,14]. Therefore, chronic IPI could maintain chronic low-grade inflammation that can increase the risk of NCD.

C-reactive protein (CRP) is a sensitive biomarker of subclinical, low-grade inflammation, it level increases during chronic disorders including metabolic ones in apparently healthy individuals [15]. High-sensitivity CRP (hsCRP) is a measurement of very low levels of plasma CRP, which provides an estimation of body inflammatory status, notably low levels of inflammation. Both biomarkers are associated with an increased risk of atherosclerosis and are recognized as independent risk factors of CVD [16–19]. As an example, high hsCRP concentrations have been shown to be associated with the risk of CVD and to predict the occurrence of myocardial infraction in apparently healthy individuals [18]. In Gabon, high prevalence of IPIs are reported with high rates among asymptomatic young people and adults who also have numerous CMDRF [20–22]. Recent surveys highlighted a high frequency of pre-high blood pressure and hyperglycemia among apparently healthy adults from urban and semi-urban settings [20]. Interestingly, although these populations carry the usual CMDRF, they are the most frequent IPI carriers and anemics [13]. Identifying the possible relationship between chronic IPI carriage and systemic inflammation would allow identification of IPIs as possible risk factors for CMD in endemic settings.

The present study assessed the level of CRP and hsCRP as biomarkers of systemic inflammation in uninfected and asymptomatic IPI carriers according to the type of parasitosis and the level of urbanization in Gabon.

## Methods

### Ethical considerations

The study protocol was authorized by the Ministry of Public Health of the Gabonese Republic and approved by the National Ethics Committee (under reference PROT N°002/2020/PR/SG/CNE). This was an observational study. The infected patients received appropriate anthelminthic or antiprotozoal treatment according to the national guidelines. Malaria, HIV and Hepatitis positive individuals were sent to the infectiology unit for appropriate case management and follow-up.

### Study sites

Volunteer were enrolled from September 2020 to November 2021 in different settlements of Gabon: Libreville (urban area) and Koula-Moutou and Bitam (both considered to be rural areas). Libreville is the capital of Gabon, with approximately 703,939 inhabitants and comprising almost half of the country’s population (Direction Générale de la Statistique 2015). Koula-Moutou, the main city of the province of Ogooué-lolo (south-eastern Gabon), is located 588 km from Libreville, with an estimated population of 25,651 inhabitants. Bitam, the main city of Ntem is located in the north of Gabon, in the province of Woleu-ntem, and comprises approximately 27,923 inhabitants.

Gabon has an equatorial climate, with temperatures ranging from 23–33°C, coupled with high humidity levels, reaching up to 80%. High rates of IPIs have been reported in the country, ranging from 68.5% to 75.2% [21,23]. Moreover, cases of schistosomiasis have also been recorded [22,24]. Data from the northern and eastern rural settlements reported high frequencies of asymptomatic IPI (53.6% and 56.6%) [22,25].

### Population and study procedures

The recruitment was performed by the teams of Centre de REcherche biomédicale en pathogènes Infectieux et Pathologies Associées (CREIPA).

Participants were individuals living in the selected urban and rural communities. During the pre-study period, the importance of the study and the benefit for the participants were presented to neighborhood chiefs in Libreville, and to village chief in rural sites. They identified community members who accompanied the research teams for the community information and awareness sessions, for leaflets and flyers distribution and community outreaches were performed. The flyers included key information, the contacts of study investigators, and locations for laboratory testing. In urban and rural sites, sensitized volunteer inhabitants were invited to come freely to the study structures or laboratories.

Before the questionnaire administration with the investigators and any testing, the participants were informed on the study objectives, procedures, risk and benefits, and that hepatitis, malaria and HIV testing will be performed prior to the inclusion. Those who accepted to be tested, signed an informed consent form before any procedures i.e. interview and stool and blood sampling for the biological analysis.

Based on volunteer’s medical history, those who did not report any clinical symptoms suggestive of an IPI (such as abdominal pain or episodes of recent diarrhea, loss of appetite, nausea or vomiting, anal itching or pruritus, with or without episodes of fever), with a normal physical examination, who did not report any antiparasitic treatment, any underlying chronic disease were considered as health individuals. They were included according to the following criteria: being aged 18 years or older, having resided for at least two years in the study area, absence of any clinical symptoms in the last six months, and providing signed informed consent. No volunteers with a serious health problem or with fever, a history of stroke, cardiovascular diseases (CDV), known chronic infection such as HIV, viral hepatitis infections, tuberculosis, or who had taken an antiparasitic treatment in the last six months were included. Those who underwent the interview and who tested positive results for HIV, hepatitis A and malaria or who were exposed to tuberculosis, were excluded.

They were interviewed, had a physical exam, and the questionnaire was completed. Then, they were given two containers for stool and urine collection, with the recommendation to return it immediately after collection or the morning after. Those who brought it were sampled for the biological testing.

In rural sites, house-hold visits were carried out every morning. The team performed the interview and physical examination of the volunteers, and they were given containers for urine and stool collection. The next day, members of the research team returned to the participant houses to collect the stool and urine containers and to perform the blood sampling.

### Sample size calculation

The sample since was calculated with the following formula: n = z^2^p(1-p)/e^2^, the sample size for this study was estimated considering the prevalence of STH (22.2%) and that of protozoa (56.4%) previously reported in urban and rural settlements of Gabon using microscopic methods, a precision of 0.05 and a 95% confidence level (z = 1.96) [21,23]. Therefore, a minimum of 378 volunteers should be included.

### Questionnaire

The sociodemographic data of the participants, age, area of residence, gender, occupation, level of school attendance were recorded on a standardized case report form.

All the biological samples were collected early in the morning and promptly transported for analysis tests performed by the study team which was located at the CREIPA for urban and semi-urban sites, at the Centre Hospitalier Régional Paul Moukambi of Koula-Moutou (CHRPM) and at the medical center of Bifolossi at Bitam.

Approximately five mL of blood samples were collected by venipuncture for C-reactive protein (CRP), high-sensitivity C-reactive protein (hsCRP) measurements, malaria, HIV, and Hepatitis B surface antigen testing. -

### Diagnosis of malaria, HIV and hepatitis B virus

Malaria infection was assessed by microscopic examination of Giemsa-stained of venous bloods drop. HIV was diagnosed using two rapid diagnosis tests according to the local algorithm: the Determine HIV early detect and the Determine HIV-1/2 Ag/Ab Combo. Hepatitis surface antigen (HBsAg) detection was performed using the Determine HBV rapid diagnostic test (Alere, France), respectively.

### Inflammatory biomarkers measurement: C-reactive protein (CRP) and high-sensitivity C-reactive protein (hsCRP)

In rural sites, dispensary and health structure did not have equipment to measure the biomarkers, CRP and hsCRP. Thus the point of care Getein1100 Immunofluorescence semi-Quantitative Analyzer which allows an on-site measurement of cardiac and inflammatory biomarkers was used (Getein Biotech, INC., Nanjing, Popular Republic of China), which is based on measurement by indirect immunofluorescence using two antibodies: an anti-human CRP monoclonal antibody coupled to the fluorescence latex located at the sample area and an anti-human CRP monoclonal antibody attached to the test line. The measurements were performed according to the manufacturer’s instructions. This semi-quantitative analyzer provides results according to cut off of biomarker values. The serum CRP concentration was considered to be elevated when the level was higher than or equal to 10 mg/L and for hsCRP, for levels higher than 3 mg/L. Thus, CRP and hsCRP levels were provided as qualitative and quantitative variables. The first 100 participants from rural sites were simultaneously tested with both the Getein 1100 and the chemistry analyzer Pentra (Horiba). Samples frozen for less than one month were used for this purpose. The sensitivity of the Getein to detect high levels of CRP and hsCRP was 98% (Agbanrin 2023, submitted).

### Diagnosis of intestinal parasitic infections

IPIs were diagnosed using four different methods as previously described [13,21,22]. The direct microscopic examination and merthiolate-iodine-formaldehyde (MIF) staining identified helminth eggs and the cystic and vegetative forms of protozoa as described elsewhere [23]. The Kato-Katz technique allowed detection and quantification of STHs and *Schistosoma* spp. on a thick slide equivalent to 41.7 mg of stool (number of eggs/g of feces). Coproculture was used for the identification of *Necator americanus* (hookworm) and *Strongyloides stercoralis* larvae. Samples were considered positive when at least one cyst, one egg, or one larva was detected.

### Statistical analysis

The data were analyzed using SPSS 20.0 software (IBM, New York, USA). The qualitative data are presented as percentages and the quantitative data were checked for the normal distribution before applying the appropriate location indicators: mean ± standard deviation for series that have a normal distribution and median and interquartile range (IQR) otherwise. The corrected Chi-square statistical test and Fischer’s exact, Wilcoxon and Mann-Whitney tests were used for the comparisons between the demographic and socioeconomic characteristics in each area.

Univariate logistic regression was performed to assess the unadjusted association between sociodemographic characteristics and the presence of IPIs; and between the presence of levels of biomarkers and age, gender, area of residence and the presence and types of IPIs. Multivariate logistic regression was used to determine the independent predictors of IPIs, high CRP and high hsCRP levels. For the evaluation of an association between presence *versus* absence of IPI and hsCRP or CRP levels with adjustment was performed in the multivariate model controlling for age, gender, study sites (urban vs. rural), level of school attendance, occupation when appropriate. This multivariate model was theory-based; thus, all predictor variables were included in the multivariate model irrespective of their statistical significance in the bivariate analysis. Adjusted odds ratio (AOR) and crude odds ratio (COR) were reported at a 95% confidence interval (CI). All *p—*values are two-tailed, and *p* ≤ 0.05 was considered statistically significant.

### Definitions

- Normal CRP level was below 10 mg/L, for hsCRP it was less than or equal to 3 mg/L.
- Coinfection: presence of at least two different types of parasites (helminth and protozoan).
- Unemployed: participants with no income and no gainful activities.
- Chronic infection: presence of any parasite in the absence of any symptoms and without antiparasitic treatment in the last six months.

## Results

### Demographic and socioeconomic characteristics of the study population

A total of 518 participants were included, 64.5% (n = 334) in urban area and 35.5% (n = 184) in rural areas. Women predominated in rural areas (59.8%, n = 110) as well as in the urban area (67.4%, n = 225).

The median age was 35 (27–46) years overall, with the inhabitants from rural areas being older than those from the urban area (42 (33–50) years vs. 32 (26–44) years; *p* < 0.01). Overall, almost half (48.5%, n = 251) of the participants were aged between 30 and 49 years old; the older ones predominated in rural area population relative to the urban area (29.3%, n = 54 vs 12.3%, n = 41, respectively; *p* < 0.01).

Two-thirds of them (68.0%, n = 352) had attended high school or secondary school, mostly from the urban area, while 19.9% (n = 103) from the rural areas did not have a professional activity. However, those with a primary education level predominated in the rural areas compared to the urban area (50.5%, n = 93 vs. 16.2%, n = 54, respectively), and a high school level predominated in the urban area relative to the rural areas (40.4%, n = 135 vs. 4.3%, n = 8, respectively; *p* < 0.01). Likewise, students predominated in the urban area population compared to the rural areas (36.8%, n = 123 vs. 3.8%, n = 7, respectively; *p* < 0.01), and the unemployment predominated in the rural area population (27.2%, n = 50 vs. 15.9%, n = 53, in the urban areas; *p* < 0.01).

### Prevalence of IPIs and relationship with sociodemographic characteristics

The overall prevalence of asymptomatic IPI was of 29.9% (n = 155). IPIs were notably more prevalent in rural settings, among individuals over 29 years of age, those with primary or secondary levels of education, and the unemployed (Table 1).

**Table 1:** Characteristics of the study population according to the presence of IPIs.

		Global	IPI	No IPI	
		N	n (%)	n (%)	*p*-value
**Area**	**Urban**	334	49 (14.7)	285 (85.3)	< 0.01
	**Rural**	184	106 (57.6)	78 (42.4)	
**Gender**	**Female**	335	93 (27.8)	242 (72.2)	0.15
	**Male**	183	62 (33.9)	121 (66.1)	
**Age groups (years)**	**18–29**	172	31 (18.0)	141 (82.0)	< 0.01
	**30–49**	251	86 (34.3)	165 (65.7)	
	**≥ 50**	95	38 (40.0)	57 (60.0)	
**School attendance**	**High school**	143	21 (14.7)	122 (85.3)	< 0.01
	**Secondary**	209	62 (29.7)	147 (70.3)	
	**Primary**	147	66 (44.9)	81 (55.1)	
	**None**	19	6 (31.6)	13 (68.4)	
**Occupation**	**Students**	130	21 (16.2)	109 (83.8)	< 0.01
	**Workers**	285	93 (32.6)	192 (67.4)	
	**Unworkers**	103	41 (39.8)	62 (60.2)	

Table 1 shows the frequency of individuals with IPI and uninfected individuals, categorized by area of residence, sex, age, school attendance and occupation.

The univariate analysis showed that residing in a rural area, being over 29 years of age, having primary or secondary school education, and being employed or unemployed constitute risk factors for IPIs, whereas residing in an urban area, being aged between 18–29 years, having completed high school education, and being a student were associated with a lower risk of IPIs (Table 2). After, the multivariate analysis, only living in a rural area was found to be a risk factor for IPIs (Table 2).

**Table 2:** Univariate and multivariate analysis of factors associated with IPIs.

		IPIs			
		COR (95%CI)	*p*-value	AOR (95%CI)	*p*-value
**Area**	**Urban**	Reference	-	Reference	-
	**Rural**	8.0 (5.2–12.0)	< 0.01	6.6 (3.2–13.8)	< 0.01
**Gender**	**Female**	Reference	-	Reference	-
	**Male**	1.3 (0.9–2.0)	0.15	1.3 (0.8–2.0)	0.32
**Age groups (years)**	**18–29**	Reference	-	Reference	-
	**30–49**	2.4 (1.5–3.8)	< 0.01	1.7 (0.9–3.4)	0.10
	**≥ 50**	3.0 (1.7–5.3)	< 0.01	1.5 (0.8–3.3)	0.32
**School attendance**	**High school**	Reference	-	Reference	-
	**Secondary**	2.5 (1.4–4.4)	< 0.01	1.2 (0.6–2.3)	0.60
	**Primary**	3.7 (1.9–6.9)	< 0.01	1.5 (0.7–3.3)	0.29
	**None**	2.7 (0.9–7.8)	0.07	0.9 (0.2–3.4)	0.88
**Occupation**	**Students**	Reference	-	Reference	-
	**Workers**	2.5 (1.5–4.3)	< 0.01	0.7 (0.3–1.6)	0.38
	**Unworkers**	3.4 (1.9–6.3)	< 0.01	0.7 (0.3–1.8)	0.47

Table 2 presents the assessment of the risk factors associated with IPI carriage. To identify the associated risk factors, a comparison was conducted using two- and multi-variable logistic regression tests, considering all characteristics of IPI carriers. The reference group was selected as the one with the lowest prevalence of IPIs. The results are presented in terms of crude odds ratio (COR) and adjusted odds ratio (AOR).

Globally, among the parasitized individuals, protozoa were detected more frequently (73.5%, n = 114/155) than helminths (40.0%, n = 62/155), the same tendency was observed in urban (81.6% (n = 40) *vs*. 22.4% (n = 11), for protozoa and STH respectively) and in rural (69.8% (n = 74) for protozoa *vs*. 48.1% (n = 51) for STHs) areas in the participants overall. Helminth–protozoa coinfection was detected in 13.5% (n = 21/155) of the infected participants, more commonly in rural areas (17.9%, n = 19) compared to urban (4.1%, n = 2) sites.

**Fig 1:**
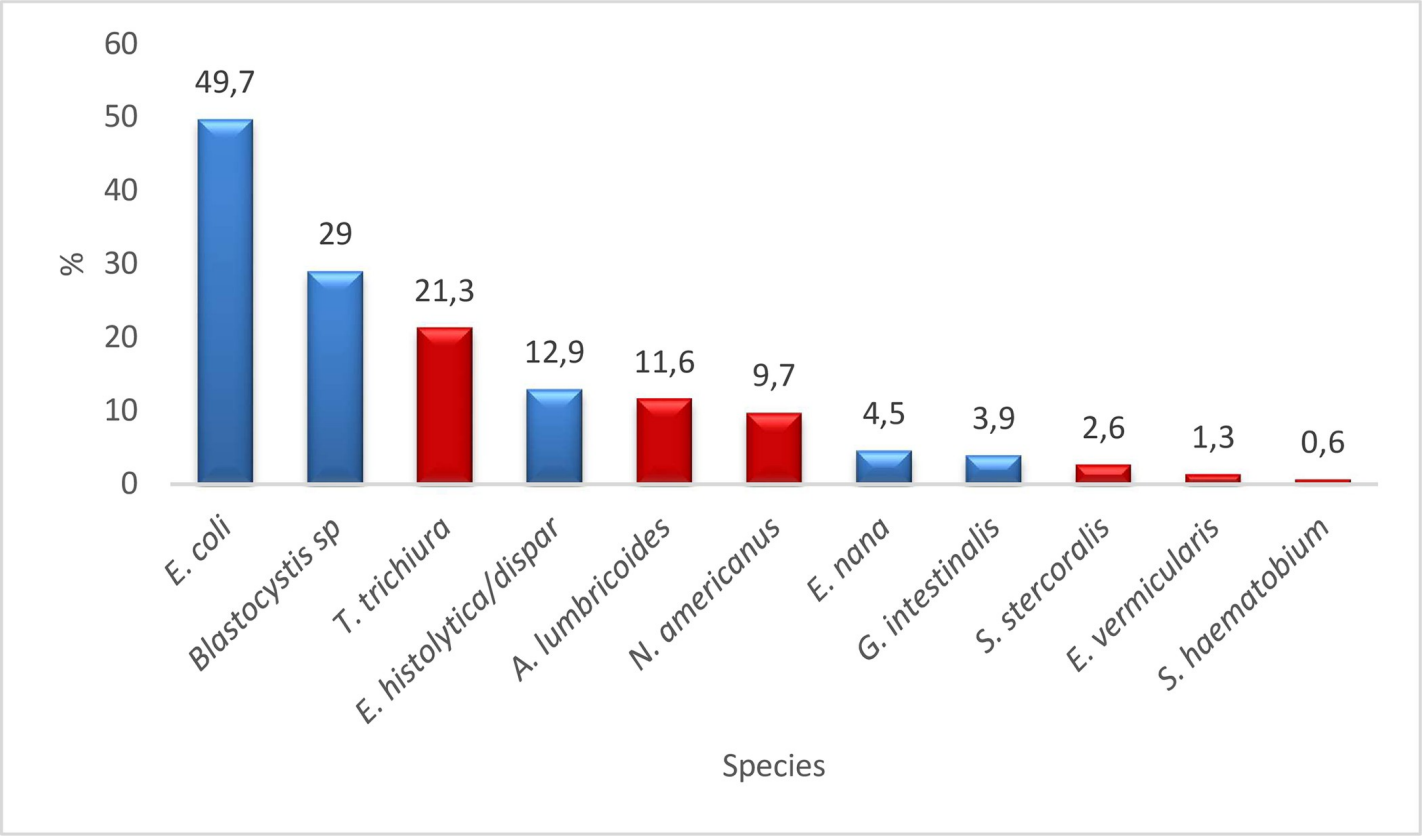
Distribution of intestinal parasite species in the study population. The blue bars of the histogram correspond to the frequency of the protozoa, the red bars to those of the helminths.

Common protozoan parasites were *Entamoeba coli* (n = 77), *Blastocystis* sp (n = 45), and *Entamoeba histolytica/dispar* (n = 20). Common STH included *Trichuris trichiura* (n = 33) and *Ascaris lumbricoides* (n = 18) (Fig 1).

### CRP and hsCRP levels according to the presence or absence of IPI

Overall, the quantitative measurement of biomarkers was performed for all the 334 participants of the urban sites and 100 from the rural ones (59 with IPI and 41 non infected ones). The median concentration of hsCRP was of 2.8 (1.4–5.2) mg/L and that of CRP was 15.1 (11.5–25.0) mg/L. In case of IPI the median values of CRP and hsCRP were 15 (13–15) mg/L and 4.2 (1.4–13.0) mg/L respectively. Among the uninfected participants, the median CRP concentration was 13.0 (11.1–14.9) mg/L and the median hsCRP concentration of 2.2 (0.4–6.1) mg/L. Differences’ between infected and non-infected participants were statistically significant (p< 0.01).

High levels of hsCRP and CRP occurred in 42.5% (n = 220) and 15.4% (n = 80) of individuals, respectively. The bivariate analysis showed that the odds of having an elevated hsCRP was 2.4 times higher in the rural as compared to urban population. High hsCRP concentrations were 1.6 to 2.1 times more frequent in individuals residing in rural areas, particularly those who were older and had IPI. Furthermore, the risk of having an elevated CRP level was higher in rural residents who were also more often parasitized (Table 3). After adjusting for sociodemographic factors, the results indicate that inhabitants from rural area were at an increased risk of presenting high levels of hsCRP (AOR 2.0 (CI 1.3–3.1), p < 0.01).

**Table 3:** Univariate analysis for factors associated with high hsCRP and high CRP.

		Elevated hsCRP (> 3 mg/L)			Elevated CRP (≥10 mg/L)		
		N (%)	COR 95% CI	*p*-value	N (%)	COR 95% CI	*p*-value
**Area**	**Urban**	116 (34.7)	Reference	-	37 (11.1)	Reference	-
	**Rural**	104 (56.5)	2.4 (1.7–3.5)	< 0.01	43 (23.4)	2.4 (1.8–3.9)	< 0.01
**Gender**	**Female**	135 (40.3)	Reference	-	49 (14.6)	Reference	-
	**Male**	85 (46.4)	1.3 (0.9–1.9)	0.18	31 (16.9)	1.2 (0.7–1.9)	0.49
**Age (years)**	**18–29**	58 (33.7)	Reference	-	21 (12.2)	Reference	-
	**30–49**	113 (45.0)	1.6 (1.1–2.4)	0.02	41 (16.3)	1.4 (0.8–2.5)	0.24
	**≥ 50**	49 (51.6)	2.1 (1.3–3.5)	0.01	18 (18.9)	1.7 (0.8–3.3)	0.14
**IPIs**	**Absent**	138 (38.0)	Reference	-	45 (12.4)	Reference	-
	**Present**	82 (52.9)	1.8 (1.3–2.7)	< 0.01	35 (22.6)	2.1 (1.3–3.4)	< 0.01
**Type of parasitosis**	**Protozoa**	44 (47.3)	1.5 (0.9–2.3)	0.10	17 (18.3)	1.6 (0.9–2.9)	0.14
	**STHs**	27 (65.9)	3.1 (1.6–6.2)	0.01	15 (36.6)	4.1 (2.0–8.3)	< 0.01
	**Co-infection**	11 (52.4)	1.8 (0.7–4.3)	0.19	3 (14.3)	1.2 (0.3–4.2)	0.80
**Number of species**	**1 protozoan**	31 (45.6)	13.4 (0.8–2.3)	0.24	14 (20.6)	1.8 (0.9–3.6)	0.08
	**2 protozoa**	9 (47.4)	1.5 (0.6–3.7)	0.42	3 (15.8)	1.3 (0.4–4.7)	0.67
	**3 protozoa**	3 (60.0)	2.4 (0.4–14.8)	0.33	0 (0.0)	0.6 (0.03–11.7)	0.76
	**4 protozoa**	1 (100.0)	4.9 (0.2–120.7)	0.33	0 (0.0)	2.3 (0.1–58.1)	0.61
	**1 STH**	23 (65.7)	3.1 (1.5–6.5)	< 0.01	14 (40.0)	4.7 (2.2–9.9)	< 0.01
	**2 STHs**	4 (66.7)	3.3 (0.6–18.0)	0.18	1 (16.7)	1.4 (0.2–12.4)	0.76
	**2 STH/protozoa**	6 (66.7)	3.3 (0.8–13.3)	0.10	0 (0.0)	0.4 (0.02–6.4)	0.49
	**3 STH/protozoa**	2 (22.2)	0.5 (0.1–2.3)	0.35	1 (11.1)	0.9 (0.1–7.2)	0.91
**Protozoan species**	***Blastocystis sp***	21 (46.7)	1.4 (0.8–2.7)	0.26	8 (17.8)	1.5 (0.7–3.5)	0.31
	***E*. *coli***	34 (43.6)	1.3 (0.8–2.1)	0.36	11 (14.1)	1.2 (0.6–2.4)	0.68
	***E*. *histolytica/dispar***	13 (65.0)	3.0 (1.2–7.8)	0.02	5 (25.0)	2.4 (0.8–6.8)	0.10
	***E*. *nana***	4 (57.1)	1.2 (0.5–9.9)	0.31	1 (14.3)	1.2 (0.3–10.0)	0.88
	***G*. *intestinalis***	6 (100.0)	21.2 (1.2–378.7)	0.04	1 (16.7)	1.4 (0.2–12.4)	0.76
**Helminth species**	***T*. *trichiura***	20 (60.6)	2.5 (1.2–5.2)	0.01	11 (33.3)	3.5 (1.6–7.8)	< 0.01
	***A*. *lumbricoides***	9 (52,9)	1.8 (0.7–4.9)	0.22	3 (17.6)	1.5 (0.4–5.5)	0.53
	***E*. *vermicularis***	0 (0.0)	0.3 (0.02–6.8)	0.47	0 (0.0)	1.4 (0.1–29.6)	0.83
	***N*. *americanus***	10 (66.7)	3.3 (1.1–9.7)	0.03	5 (33.3)	3.5 (1.2–10.8)	0.03
	***S*. *stercoralis***	4 (100.0)	14.7 (0.8–274.3)	0.07	1 (25.0)	2.4 (0.2–23.1)	0.46

This table shows the frequency of elevated CRP and hsCRP concentrations as a function of the areas of residence, the sex, the age, the presence of IPI and according to the different species of parasites. On the other hand, the ORs were calculated by bivariate analysis. STH: soil–transmitted helminth, *E*. *coli*: *Entamoeba coli*, *E*. *histolytica/dispar*: *Entamoeba histolytica/dispar*, *E*. *nana*: *Endolimax nana*, *G*. *intestinalis*: *Giardia intestinalis*, *T*. *trichiura*: *Trichuris trichiura*, *A*. *lumbricoides*: *Ascaris lumbricoides*, *E*. *vermicularis*: *Enterobius vermicularis*, *N*. *americanus*: *Necator americanus*, *S*. *stercoralis*: *Strongyloides stercoralis*.

STH carriers were at higher risk of having high hsCRP (COR 3.1 (CI 1.6–6.2), *p* < 0.01) and high CRP (COR 4.1 (CI 2.0–8.3), *p* < 0.01) levels (Table 3). A trend toward an association between protozoan carriage and high hsCRP (COR 1.5 (CI 0.3–2.3), *p* = 0.10) and high CRP levels was also observed (Table 3).

Medians of CRP (15.7 (12.1–24.2) mg/L) and hsCRP (6.0 (2.2–19.0) mg/L) levels were significantly higher in STH carriers compared to protozoan (14.6 (13.9–15.8) mg/L for CRP level and 3.2 (1.0–7.4) mg/L for hsCRP) carriers, to the STH-protozoa (13.0 (11.7–15.0) mg/L for CRP level and 3.7 (1.8–9.6) mg/L for hsCRP) carriers and the uninfected persons (p < 0.01).

All carriers of *Giardia intestinalis* and *Strongyloides stercoralis* had elevated hsCRP levels. However, bivariate analyses indicated that the presence of *Entamoeba histolytica/dispar* and *Giardia intestinalis* were significantly associated with a higher frequency of a high hsCRP concentration compared to the absence of IPI. Participants infected by *Trichuris trichiura* and hookworm had significantly higher hsCRP concentrations (Table 3). Likewise, *Trichuris trichiura* and *Necator americanus* carriages were associated with a high CRP concentrations (Table 3).

### Association between CRP, hsCRP and sociodemographic characteristics in parasitized participants

Male participants tended to have a higher risk of elevated hsCRP and CRP levels when infected in comparison to females (Table 4). Residing in remote regions posed a risk of elevated CRP levels. Advancing age, however, was not found to elevate the risk of inflammation in parasitized individuals (Table 4).

**Table 4:** Bivariate and Multivariate analysis of factors associated with high inflammatory biomarkers among the IPI—infected participants.

		Elevated hsCRP					Elevated CRP				
		N (%)	COR 95% CI	*p—value*	AOR 95% CI	*p—value*	N (%)	COR 95% CI	*p—value*	AOR 95% CI	*p—*value
**Area**	Urban	22 (44.9)	Reference	-	Reference	-	6 (12.2)	Reference	-	Reference	-
	Rural	60 (56.6)	1.6 (0.8–3.2)	0.18	0.7 (0.3–1.4)	0.28	29 (27.4)	2.7 (1.0–7.0)	0.04	2.4 (0.9–6.5)	0.08
**Gender**	Female	44 (47.3)	Reference	-	Reference	-	16 (17.2)	Reference	-	Reference	-
	Male	38 (61.3)	1.8 (0.9–3.4)	0.09	1.7 (0.9–3.4)	0.10	19 (30.6)	2.1 (1.0–4.6)	0.05	2.0 (0.9–4.4)	0.07
**Age groups**	18–29	16 (51.6)	Reference	-	Reference	-	5 (16.1)	Reference	-	Reference	-
	30–49	43 (50.0)	0.9 (0.4–2.1)	0.88	1.2 (0.5–2.8)	0.68	19 (22.1)	1.5 (0.5–4.4)	0.48	1.3 (0.4–3.8)	0.70
	≥ 50	23 (60.5)	1.4 (0.5–3.7)	0.46	0.8 (0.3–2.4)	0.73	11 (28.9)	2.1 (0.6–7.0)	0.22	1.5 (0.4–5.3)	0.50

## Discussion

The present study documented the CRP and hsCRP levels in apparently healthy residents from urban and rural areas of Gabon according to the carriage of intestinal parasites.

The overall prevalence of asymptomatic IPIs found in these populations was lower than in previous reports in other urban and rural areas of Gabon [21–22]. The higher risk of IPIs, mainly STHs in rural sites, is not surprising. STHs are known to be prevalent in areas where certain existing conditions such as poverty, low level of hygiene and sanitation, and limited access to safe drinking water, favor their spread. The prevalence of asymptomatic intestinal parasitic infections (IPI) was lower compared to previous studies conducted in Gabon and elsewhere [21,23,26,27]. The low frequency of IPIs in urban inhabitants which represented almost two thirds (64.5%) of the study population and among which the IPI prevalence was 1.8-fold lower than that of residents in rural sites (35.5% vs. 64.5%), could be in cause. Secondly, the rate of self-medication with antiparasitic drugs among adults compared to children who were most represented in previous surveys, would also explain this discrepancy [21,23]. Furthermore, only one stool sample was taken per participants, it is known that the parasite density may vary throughout the course of infection for some IPI. Nevertheless, four different microscopic methods were used, their combined sensitivities for parasite detection would have reduced the risk of missing several low parasite density carriers. Compared to high-income countries, high DALYs due to NCD attributed to ID have been reported in LMIC [28]. Thus, while the current westernized lifestyle of inhabitants of SSA urban cities and capitals can partly explain the increase of NCD, this is not the case in rural and remote settlements, where populations live under different conditions with traditional lifestyles.

This study is one of the first to analyze a possible relationship between systemic inflammation, which is a recognized risk factor for CVD, and the presence of IPIs in Central Africa. Our hypothesis that chronic intestinal parasitism can induce a chronic inflammation was tested by measurement of CRP and hsCRP levels, which are biomarkers and indicators of low-grade inflammation in chronic infection [29]. CRP and hsCRP serum levels are independently and significantly associated with the risk of developing ischemic cardiovascular diseases [16]. Moreover, high hsCRP was shown to be associated with the risk of CD and to predict the occurrence of myocardial infarction in healthy individuals [18].

An association between IPI carriage and the studied biomarkers of systemic inflammation was found Consistent with data reported elsewhere, the age of the participants was no found to be a significant risk factor for systemic inflammation [30]. The trends observed in the bivariate analysis is probably due to the predominance of participants who were aged more than 45 years old in the rural sites, the highest levels of IPIs was also recorded among them. The higher exposure and frequency of STH in rural areas justify the higher risk of elevated CRP and hsCRP concentrations when living in this area. These parasites induce mechanical and chemical damage, mucosal inflammation, and host immune responses, which are leading causes of long-term inflammation, thereby representing potential drivers of NCD such as heart, lung, renal, and gastrointestinal diseases as well as cancers [31,32]. Accordingly, the present results, suggest that asymptomatic carriage of helminths is associated with systemic low grade inflammation. For instance, some hookworms cause chronic iron-deficiency anemia; and iron deficiency has been reported to be associated with an increase in hsCRP or other inflammatory markers among healthy adults [33,34]. Through egg migration-induced inflammation, egg embolization, and granuloma development, *Schistosoma* species are causes of cancer, induce renal diseases or pulmonary hypertension which can cause right ventricular failure [35]. Heavy trichuriasis causes chronic infiltration of inflammatory cells in the colon of infected children as well as iron-deficiency anemia [36]. Consistent with this, *Schistosoma* spp., hookworm, and *Trichuris trichiura*, chronic carriage was found associated with increased levels of CRP and hsCRP and thus certainly with low-grade chronic inflammation.

Strongyloidiasis appears to attenuate the systemic inflammation during coinfection or comorbidities [37]. In the present study, asymptomatic *Strongyloides stercoralis* carriers had a high hsCRP levels; further investigations in this regard should be performed.

Regarding protozoa, hsCRP levels were also high in case of pathogenic *Entamoeba histolytica* and *Giardia intestinalis* carriage. These pathogenic species, as well *Blastocystis spp*., can cause inflammation [8]. Globally, enteropathogens reduce iron absorption and increase systemic inflammation [38]. Slight increase of hsCRP in obese children infected with *G*. *intestinalis* but with a trend towards lower metabolic syndrome were reported by Caudet et *al*. [39]. Several co-factors could influence the direction of an association between parasites and inflammation. Indeed, it is suggested a reduction or absence of inflammation with *Giardia* infection, a decrease in subsequent rates of diarrhea and fever, as well as lower systemic levels of C-reactive protein in children [40,41]. *G*. *intestinalis* was shown to alter the abundance of gut microbiome bacteria, a marker of gut dysbiosis, the presence of this parasite in the intestine was also associated with an excess of fecal calprotectin levels a marker of intestinal inflammation [42]. Considering that a chronic disruption of the gut barrier can lead to translocation of microbial components into the body, producing systemic, low-grade inflammation, the role of *Giardia* and other protozoa on inflammation should be considered in the context of multiple enteropathogens impact on the microbiome function [43]. Enteric bacteria were also show to be associated with inflammation [38].

To limit other causes of inflammation, participants with known chronic infectious diseases and known chronic inflammatory diseases were not included, but the asymptomatic carriage of other enteropathogens was not studied. Therefore, as suggested by others, the pathogenic or beneficial impact of *G*. *intestinalis* on inflammation should be further investigating by integrating various nutritional, microbial, metabolic and immune factors [44,45]. Th2 response should be characterized in chronic protozoan carriers and association with dysbiosis and low-grade inflammation should also be investigated.

This study has some limitations. First, although participants were asymptomatic and free of previous antiparasitic treatment, a recent instead of chronic IPI could not be formally excluded. Submicroscopic parasite carriers were not identified, in the absence of molecular diagnosis of IPI, therefore, they would have been misclassified as uninfected participants. IPIs were detected by using four different microscopic tests, this procedure increases the sensitivity of the microscopic detection which involved only one or two microscopic tests most of the time. Although information and awareness campaigns, it is not easy to obtain several samplings in adult communities who do not, spontaneously, provide even one sample when they are not sick. Our results should be confirmed with larger studies which will include the analysis of several stool samples per individual and the molecular detection of IPI to increase the sensitivity of low parasite density detection. Nevertheless, molecular tools and adequate stool sample storage conditions are lacking in most of the IPI endemic areas, where such study could be reproduced. Secondly, the detection of other enteropathogens such as bacteria and fungi, either as invasive pathogens or just colonized ones, was not performed. The analysis of the gut microbiota of the study participants is ongoing. Biomarkers were measured in apparently healthy individuals residing in the same urban or rural city, with comparable living conditions including housing and type of diet, within each site. Thus, they should share comparable levels of exposure to some NCD risk factors. Moreover, the analysis performed here are comparable to those in other reports regarding the relationship between infectious diseases or agents and our studied inflammation biomarkers [38,46].

It is important to note that there is a paucity of previous reports on this topic, especially in adults who carry the double burden of asymptomatic IPI and a higher risk of NCD.

## Conclusion

The present study suggests an association between STH, pathogenic protozoan intestinal carriage and low-grade inflammation, which is risk factor for NCD, mainly cardiovascular diseases. In rural settlements, permanent exposure to STH may contribute to the increased risk of high hsCRP and high CRP levels among inhabitants. Molecular-based approached infectious agent detection along with nutritional and immune factors should be included in longitudinal studies to better elucidate the negative or positive impact of overall and/or specific enteropathogens on dysbiosis and low-grade chronic inflammation.
